# Comparisons of Portable Sleep Monitors of Different Modalities: Potential as Naturalistic Sleep Recorders

**DOI:** 10.3389/fneur.2016.00110

**Published:** 2016-07-15

**Authors:** Masahiro Matsuo, Fumi Masuda, Yukiyoshi Sumi, Masahiro Takahashi, Naoto Yamada, Masako Hasegawa Ohira, Koichi Fujiwara, Takashi Kanemura, Hiroshi Kadotani

**Affiliations:** ^1^Department of Psychiatry, Shiga University of Medical Science, Otsu, Japan; ^2^Faculty of Education, Shiga University, Hikone, Japan; ^3^Department of Systems Science, Kyoto University, Kyoto, Japan; ^4^Department of Sleep and Behavioral Sciences, Shiga University of Medical Science, Otsu, Japan

**Keywords:** portable sleep monitors, activity recorders, single channel EEG, polysomnography, sleep estimation

## Abstract

**Background:**

Humans spend more than one-fourth of their life sleeping, and sleep quality has been significantly linked to health. However, the objective examination of ambulatory sleep quality remains a challenge, since sleep is a state of unconsciousness, which limits the reliability of self-reports. Therefore, a non-invasive, continuous, and objective method for the recording and analysis of naturalistic sleep is required.

**Objective:**

Portable sleep recording devices provide a suitable solution for the ambulatory analysis of sleep quality. In this study, the performance of two activity-based sleep monitors (Actiwatch and MTN-210) and a single-channel electroencephalography (EEG)-based sleep monitor (SleepScope) were compared in order to examine their reliability for the assessment of sleep quality.

**Methods:**

Twenty healthy adults were recruited for this study. First, data from daily activity recorded by Actiwatch and MTN-210 were compared to determine whether MTN-210, a more affordable device, could yield data similar to Actiwatch, the *de facto* standard. In addition, sleep detection ability was examined using data obtained by polysomnography as reference. One simple analysis included comparing the sleep/wake detection ability of Actiwatch, MTN-210, and SleepScope. Furthermore, the fidelity of sleep stage determination was examined using SleepScope in finer time resolution.

**Results:**

The results indicate that MTN-210 demonstrates an activity pattern comparable to that of Actiwatch, although their sensitivity preferences were not identical. Moreover, MTN-210 provides assessment of sleep duration comparable to that of the wrist-worn Actiwatch when MTN-210 was attached to the body. SleepScope featured superior overall sleep detection performance among the three methods tested. Furthermore, SleepScope was able to provide information regarding sleep architecture, although systemic bias was found.

**Conclusion:**

The present results suggest that single-channel EEG-based sleep monitors are the superior option for the examination of naturalistic sleep. The current results pave a possible future use for reliable portable sleep assessment methods in an ambulatory rather than a laboratory setting.

## Introduction

Sleep is a physiological phenomenon that occupies more than one-fourth of the human lifespan. Accordingly, sleep disorders have been linked to various medical conditions, including cardiovascular disease, diabetes, and dementia (1–3). Such sleep disorders include not only frequently observed sleep apnea but also circadian rhythm disorders and insomnia. However, the underlying function of sleep remains uncertain partially due to the heterogenic level of sleep within each sleep session (4). Furthermore, it has been stressed that sleep quality and quantity vary significantly on a nightly basis, depending on the environment or workload on the preceding day (5–7).

The current gold standard for sleep examination is polysomnography (PSG), in which patients are required to spend one night in a specialized room. During PSG examination, sleep features are recorded by various sensors, including multichannel electroencephalography (EEG) (8). This system requires well-trained technicians in addition to a highly sophisticated EEG system that allows the recording of subtle electrical activity in the human body. However, the likelihood of capturing rare sleep-related events in a single examination session is low. Accordingly, in a clinical setting, patients often report sleep symptoms that only occur once in a few nights.

In addition to problems inherent to PSG, a recent study has indicated the importance of naturalistic sleep examination in daily life, instead of in a sleep examination room. A further study reported that even multiple sleep assessments in a PSG examination room might not accurately represent naturalistic sleep quality (9). In conjunction with the well-established first-night effect (10), these studies indicate that single-night sleep assessment methods might not adequately represent the nature of sleep in daily life. Therefore, a convenient method for the assessment of sleep quality in daily life might provide an ideal solution to this problem.

Several different devices capable of portable sleep assessment are currently available. The majorities of these devices utilize body movements or abridged EEG signals as an indicator of sleep status.

Activity-based sleep monitors, including the frequently used Actiwatch (Actiwatch2, Philips Respironics, Amsterdam, Netherlands), are also used as daytime activity monitors, because they are small devices that do not hinder activities of daily life. Because of this advantage, such devices are often preferred to detect unpredicted sleep fall. Devices of this kind have undergone significant improvement in the past few decades to improve and expand their utility (11–14). In the current era of wearable devices, activity sensors have become increasingly economical and new devices have been developed (15, 16), including the MTN-210 (Kissei Comtec, Nagano, Japan). Both research level and consumer level devices have become attractive options for the study of naturalistic sleep status.

In sharp contrast to the widely used activity-based sleep monitors, EEG-based sleep monitors have a relatively short history of use (17, 18). Since EEG electrodes are attached to the head during recording, this typically limits their use to nighttime. However, sleep architecture is only directly observable through EEG activity; therefore, EEG-based monitors can potentially be used for the assessment of naturalistic sleep quality. In addition, the simpler mechanism of EEG is preferred for the recording of naturalistic sleep, since untrained subjects will be required to use this method in their home. Accordingly, single channel EEG has recently attracted attention (19–21), although its reliability has not been compared to that of activity-based sleep recorders. To address this question, an EEG recorder Sleep Scope (Sleepwell, Osaka, Japan) was used in the present study, whose prototype device has been previously validated in patients with sleep disorders (22).

Since portable devices are more economical and less complex than PSG, and assessing naturalistic sleep is of great importance, it will be of interest for both clinicians and patients if the physiological validity and reliability of these portable devices are established. However, the reliability of not only newly developed devices but also currently used devices remains unclear. Therefore, the present study aimed to address this question by conducting cross-modal comparisons of the sleep monitors previously discussed.

## Materials and Methods

### Participants

Twenty-two healthy volunteers participated in this study. Due to an Actiwatch malfunction and a data download failure in an MTN-210, two participants were excluded from analysis. The total participants therefore included 11 males and 9 females (age range 19–24 years; mean age 20.70 ± 0.39). Eighteen participants were undergraduate students, and two were graduate school students. None of them were obese (body mass index: 20.68 ± 0.44 kg/m^2^) or pregnant. The Mini International Neuropsychiatric Interview (23) was used to screen past medical history, and an additional interview by experienced psychiatrists found that none of the participants had a record of psychiatric or sleep disorders.

All study procedures were approved by the Ethics Committee of Shiga University of Medical Science.

Written informed consent was obtained from all participants, and the study was performed in accordance with the Declaration of Helsinki.

### Actigraphy Analysis

Actiwatch and MTN-210 were used for activity-based sleep recordings. Participants were required to wear Actiwatch devices around the non-dominant wrist. To compare the inter-device differences in activity data, one MTN-210 was clipped to the Actiwatch wristband to ensure these two devices were exposed to the same range of movement. Moreover, participants were required to wear another MTN-210 on the front side of the trunk, by clipping it to waist belt or to the edge of the trousers/pants. Recording started at 8:00 a.m. on day 1, and PSG recording was performed on the night of day 7. Actigraphy recordings were stopped at 8:00 a.m. in the morning after PSG recording was completed. All devices were configured to record activity every 2 min. A 2-min epoch was used, since this time window is often used to save memory space when long-term activity logging is required. Since perfect synchronization was required between PSG and the activity monitors for epoch-by-epoch evaluation, all devices were synchronized to one timeserver through the Internet. Furthermore, synchronization was visually confirmed by tracing activity bursts intentionally evoked prior to PSG recording. By using these collection protocols, 5040 2-min epochs were collected before and during PSG examination. Data were extracted from MTN-210 devices through an NFC interface (PaSoRi, RC-S380, Sony Corporation, Japan) using SleepSign Act software (Kissei Comtec, Nagano, Japan). For sleep/wake detection from MTN-210 data, default settings in SleepSign Act were used, in which sleep detection followed the previously reported algorithm (24). Data were extracted from Actiwatch devices using Actiware 6.0.1 (Philips Respironics, Amsterdam, Netherlands) through a designated device cradle. To determine the method-dependent changes in sleep/wake detection, we used three different Actiware thresholds; low (20), medium (40), and high (80). This software scores epochs by applying these thresholds to the weighted-moving-average of activity data. This algorithm has been validated to PSG data (25).

### SleepScope Analysis

SleepScope (SS) is a single channel portable EEG device from SleepWell (Osaka, Japan). SS recordings were conducted on the last night concurrent to PSG recording. Both the method and analysis of the SS recordings are described in detail elsewhere (26). But briefly, one SS electrode was placed in the middle of forehead and the other electrode on left mastoid. In addition, the data obtained by SS were forwarded to cloud services (SEAS-G, Sleepwell, Osaka, Japan), in which spectral analysis of the EEG data was performed for every 30-s epoch, and they are classified into five sleep stages: wake, REM, stage 1, stage 2, and stage 3. Stage information was provided with the time stamp, and the EEG trace is also available for download. These timing data allowed us to synchronize SS results with other data set used in this study. This service is approved by Japanese Medical Device Certification (225ADBZX00020000).

### Sensitivity Analysis of Activity Sensors

To investigate the differential sensitivities of MTN-210 and Actiwatch, the devices were exposed to the same activity by placing them on an iron bar that was loosely attached to the ridge of a shaker plate (BR-13UM, TAITEC, Japan). The shaker was set at five discrete speeds (0, 50, 75, 100, and 150 rpm) for 5 min, and the corresponding count recordings were compared.

### PSG Recordings

Polysomnography recordings were performed using an Alice-5 system (Respironics Inc., Murrysville, PA, USA) with the following set of measurements: four-electrode scalp-encephalography (C3–A2, C4–A1, O1–A2, and O2–A1), two-electrode electrooculography (placed near edge of the eyes), electrocardiography, and electromyography, in addition to sensors for the detection of oral/nasal airflow, and chest/abdominal movements. PSG data were recorded online by Alice Sleepware (version 2.8, Respironics Inc., Murrysville, PA, USA). An experienced PSG specialist, who was blind to participants’ conditions, scored sleep stages visually.

In order to match the 30-s PSG epochs to the 2-min actigraphy epochs, the most frequent sleep stage scored by PSG within the corresponding 2-min actigraphy epoch was designated as the representative sleep stage of the 2-min epoch.

### Statistical Analysis

A paired Student’s *t*-test was performed where appropriate. One-way analysis of variance (ANOVA) was used to compute significance between more than three groups, and a *post hoc* test with Tukey correction was applied to test individual comparisons. All statistical analyses were performed using PRISM-6 software (GraphPad, La Jolla, CA, USA). Data are presented as mean ± SE, unless otherwise stated.

## Results

### Difference and Preference of Activity Monitors

**Figure 1:**
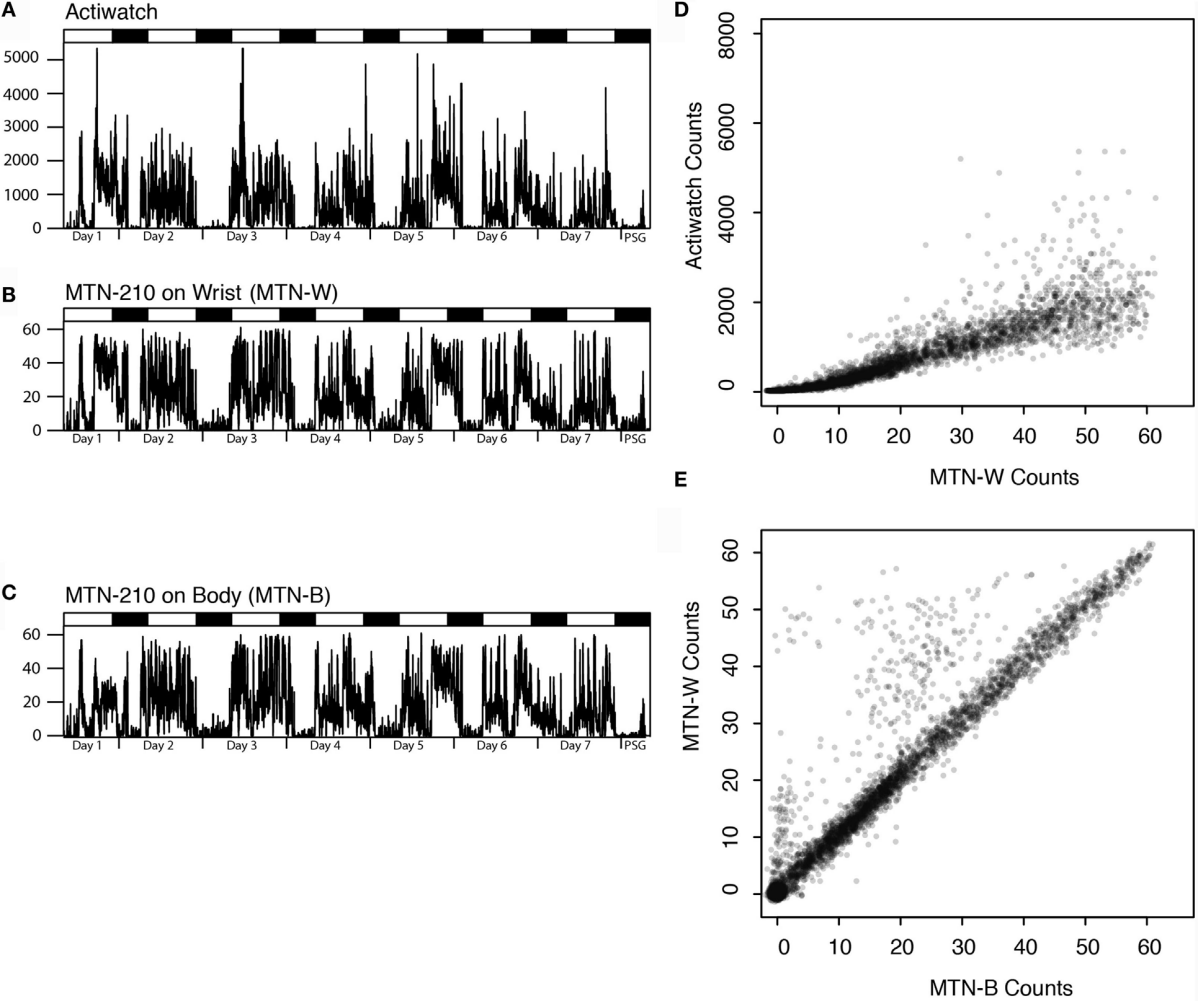
**Representative actigraphs from Actiwatch, MTN-210 on the wrist, MTN-210 on the body, and corresponding analysis**. Actigraphs of 7 days prior to and during PSG are shown, where the days and time when PSG was conducted are shown on the *X*-axis, and the activity counts for every 2 min **(A–C)** are shown on the *Y*-axis. Correlation of the activity between MTN-W and Actiwatch is compared by scatter plot **(D)** or MTN-B and MTN-W **(E)**. MTN-B, MTN-210 on the body; MTN-W, MTN-210 on the wrist; PSG, polysomnography.

First, the differences between the two activity recorders were examined by comparing the wrist data from Actiwatch (ACT-W) and MTN-210 (MTN-W). In addition, the effects of recording site were assessed by placing another MTN-210 on the body trunk (MTN-B). Figure 1 displays the representative 7-day actigraphs from one participant and the corresponding analysis. We conducted following analysis using all 7-day data.

Good agreement was found between MTN-W and ACT-W data (Spearman’s rank correlation: *r* = 0.97 ± 0.00, *P* < 0.01, *n* = 20, Figure 1D), although Actiwatch counts were two orders of magnitude higher than the MTN-W counts (max counts: MTN-W, 61.65 ± 0.17; ACT-W, 7337.05 ± 892.51; *n* = 20), suggesting a considerable difference in the sensitivity resolution of each device.

**Figure 2:**
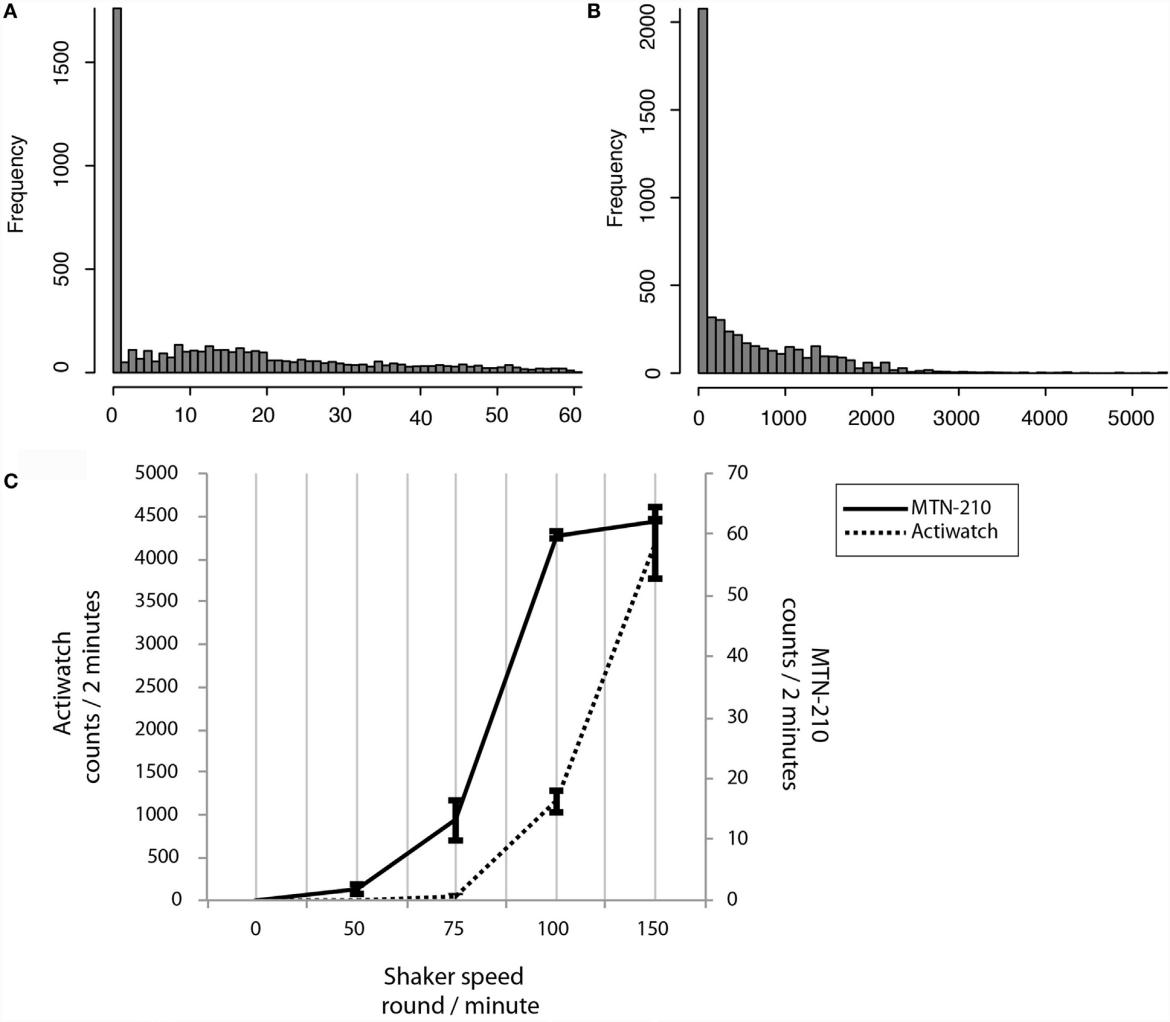
**Sensitivity preferences and differences between MTN-W and Actiwatch**. Distributions are shown by frequency on the *Y*-axis, and corresponding activity strength is shown on the *X*-axis **(A,B)**. Sensitivity preferences were compared. The shaker speed is indicated on the *X*-axis, and the corresponding counts are displayed on the *Y*-axis **(C)**. MTN-W, MTN-210 on the wrist.

By activity sensor sensitivity analysis, it was demonstrated that the activity count recordings were not linearly correlated to the shaker speed, and that the ranges of ultra-sensitivity were identified [75–100 rpm (roughly corresponding to 10–60 counts/2 min) for MTN-210 and over 100 rpm (roughly corresponding to 1000 counts/2 min) for Actiwatch (Figure 2C)].

Intriguingly, real-life activity data distributions were extremely low compared to these superior sensitivity ranges. For example, 61.85 ± 1.65% of activity was lower than 10 counts/2 min for MTN-210, and 97.06 ± 0.28% of activity was lower than 1000 counts/2 min for Actiwatch (Figures 2A,B).

On comparing the placements, a strong correlation was identified between activity data from MTN-W and MTN-B, despite sporadic higher counts owing to hand activity from the MTN-W placement site (Spearman’s rank correlation: *r* = 0.87 ± 0.03, *P* < 0.01, *n* = 20, Figure 1E). These data indicated that a resembling activity pattern could be obtained independent of the placement on the body.

### Comparison of Portable Monitors as Sleep Recorders

Next, the sleep/wake detection abilities of activity-based and single-channel EEG-based sleep monitors were compared. Using the software provided by the manufacturers, sleep parameters were assessed using the 2-min epoch data. For the analysis of Actiwatch data, wake detection thresholds were set to 80, 40, or 20, and these conditions were referred to as Act80, Act40, and Act20, respectively.

The average duration of recordings was 477.70 ± 3.79 min, and the average sleep duration as assessed by PSG was 419.10 ± 10.88 min (range: 286–462 min), suggesting that all participants slept for an adequate time during the analysis.

**Figure 3:**
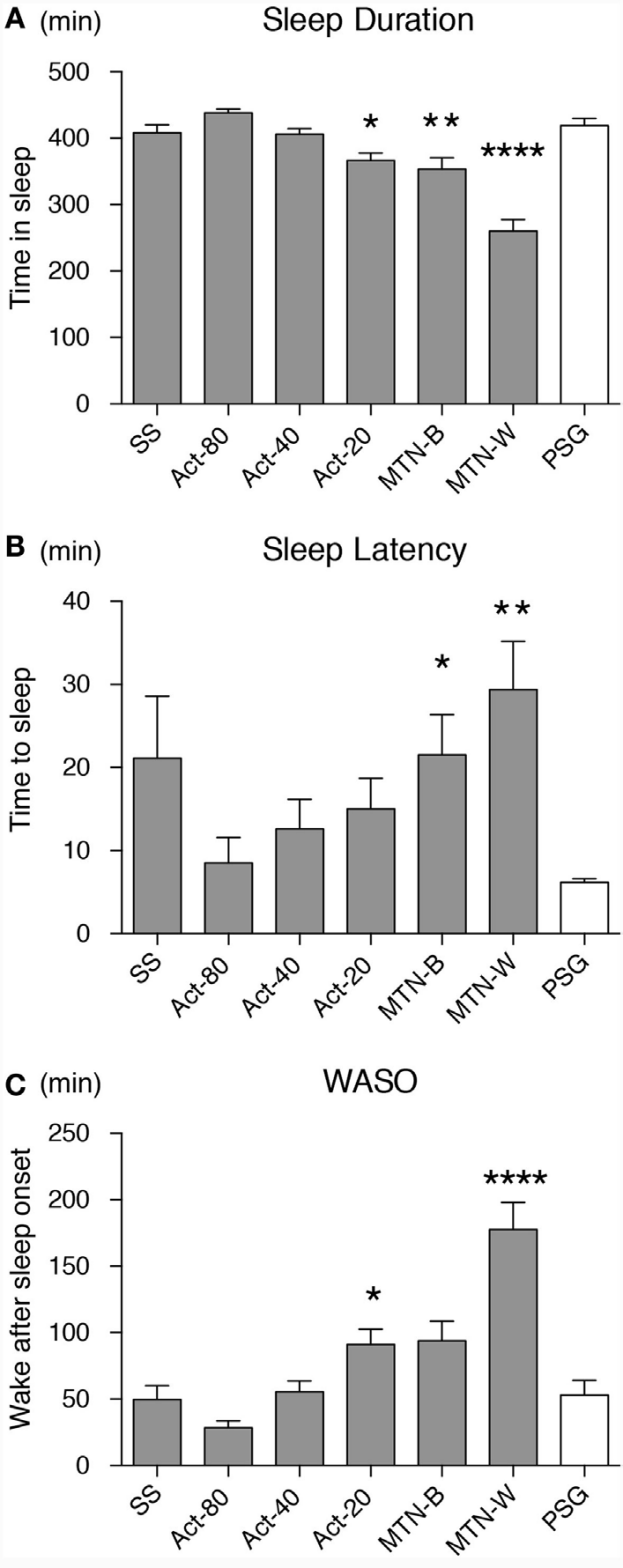
**Sleep parameters were compared among the devices/conditions**. Sleep duration **(A)**, sleep latency **(B)** and wake after sleep onset **(C)** estimations by all the devices/conditions are compared. Conditions are displayed on the *X*-axis, and parameter values on the *Y*-axis. White box shows data from PSG as a reference. Asterisks show levels of significance: *P *<* 0.05, **P *<* 0.01, ***P *<* 0.001. PSG, polysomnography.

**Figure 4:**
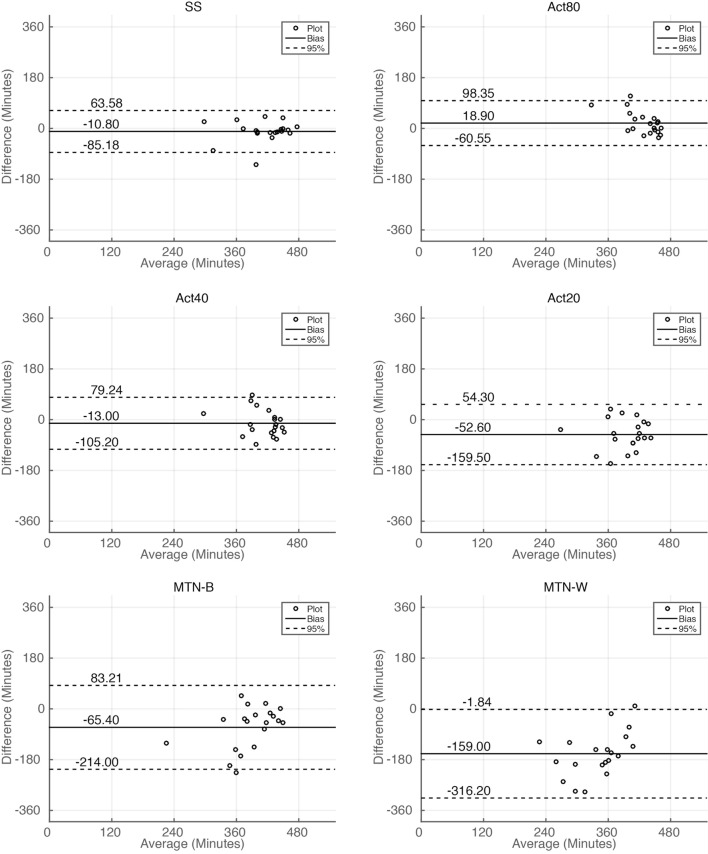
**Bland–Altman plot analysis of sleep duration estimations**. Bland–Altman plot for sleep duration estimations by all the methods. Horizontal solid lines represent the means of the differences, and dashed lines represent 95% limits of agreement.

Sleep durations estimated by SS, Act80, Act40, Act20, MTN-B, and MTN-W were 408.30 ± 11.99, 438.00 ± 5.76, 406.10 ± 8.32, 366.50 ± 11.33, 353.70 ± 16.85, and 260.10 ± 17.65 min, respectively (Figure 3A). These data indicated that Act20, MTN-B, and MTN-W estimated significantly shorter sleep durations than PSG (*P* < 0.05 for Act20, *P* < 0.01 for MTN-B, and *P* < 0.0001 for MTN-W, one-way ANOVA after Dunnett’s correction). This finding is also supported by Bland–Altman analysis, where SS, Act80, and Act40 featured bias less than 20 min, whereas others demonstrated larger bias (Figure 4; Table 1). Correlation analysis further demonstrated that SS estimations featured a strong correlation (*r* = 0.73, *P* < 0.01), while Act80 featured a modest correlation with PSG (*r* = 0.55, *P* < 0.05).

**Table 1:** **Sleep parameters were compared among the devices/conditions**.

	Bland–Altman	Correlation analysis		
		Bias (95% limits of agreement)	Pearson *r*	*P* (two-tailed)	
**(A) Sleep duration**				
SS	−10.8 (−85.18 ± 63.58)	0.7286	0.0003	***
Act80	18.9 (−60.55 ± 98.35)	0.5534	0.0114	*
Act40	−13 (−105.2 ± 79.24)	0.4246	0.062	ns
Act20	−52.6 (−159.5 ± 54.3)	0.3975	0.0826	ns
MTN-B	−65.4 (−214 ± 83.21)	0.3131	0.1789	ns
MTN-W	−159 (−316.2 ± −1.842)	0.2821	0.2281	ns
**(B) Sleep latency**				
SS	14.9 (−49.54 ± 79.34)	0.3597	0.1193	ns
Act80	2.3 (−24.33 ± 28.93)	0.1851	0.4347	ns
Act40	8.5 (−63.3 ± 80.3)	0.2434	0.3011	ns
Act20	8.8 (−22.62 ± 40.22)	0.2518	0.2841	ns
MTN-B	15.3 (−26.87 ± 57.47)	0.1626	0.4935	ns
MTN-W	23.2 (−27.25 ± 73.65)	0.06577	0.7829	ns
**(C) WASO**				
SS	−3.4 (−83.33 ± 76.53)	0.6397	0.0024	**
Act80	−24.6 (−106.6 ± 57.38)	0.5259	0.0172	*
Act40	2.5 (−93.77 ± 98.77)	0.3694	0.1089	ns
Act20	38.2 (−76.5 ± 152.9)	0.3204	0.1685	ns
MTN-B	40.9 (−96.4 ± 178.2)	0.2793	0.2331	ns
MTN-W	124.5 (−59.14 ± 308.1)	0.2198	0.3517	ns

*Bland–Altman analysis and correlation analysis were conducted against estimations by various methods. Panel (A) shows result for sleep duration, (B) for sleep latency, and (C) for WASO*.

*WASO, wake after sleep onset*.

Sleep latency as defined by PSG was 6.20 ± 0.43 min. Sleep latency estimations by SS, Act80, Act40, Act20, MTN-B, and MTN-W were 21.1 ± 7.50, 8.50 ± 3.09, 12.60 ± 3.54, 15.00 ± 3.67, 21.50 ± 4.86, and 29.40 ± 5.77 min, respectively (Figure 3B). MTN-B and MTN-W estimated a significantly longer sleep latency (*P* < 0.05 for MTN-B and *P* < 0.01 for MTN-W). Compared to the short duration of sleep latency, bias related to these methods was relatively large (Table 1). All methods had longer bias than the actual length of sleep latency (6.20 min) except for Act80, demonstrating that these methods can overestimate sleep latency by double. In addition, none of the sleep latency estimations demonstrated a correlation with PSG results.

Similarly, wake after sleep onset (WASO) was examined in the present study (Figure 3C). PSG results indicated that WASO was 53.00 ± 10.98 min, while SS, Act80, Act40, Act20, MTN-B, and MTN-W estimated WASO at 49.60 ± 10.48, 28.40 ± 5.23, 55.50 ± 8.13, 91.20 ± 11.46, 93.90 ± 14.65, and 177.50 ± 20.42 min, respectively. This analysis found that estimations by Act20 and MTN-B were significantly longer than additional methods (*P* < 0.05 for Act20 and *P* < 0.0001 for MTN-W). Bland–Altman analysis support that SS was suitable for WASO estimation, as it had small bias (−3.4 min) with strong correlation (*r* = 0.64, *P* < 0.01) followed by Act80 with bias less than half an hour (−24.6 min) and moderate correlation (*r* = 0.53, *P* < 0.05).

The difference in estimated sleep parameters suggested that sleep/wake delineation differed significantly depending on the choice of device or threshold. To examine the ability of each method to delineate sleep/wake epochs, the sensitivity and specificity of each method was assessed. The analysis identified superior performance using the SS method, as its sensitivity was 92.43 ± 1.83% and specificity was 69.69 ± 4.95% (Table 2). Similar sensitivity (93.00 ± 0.81%) was found in Act80, but this high sensitivity was achieved at the cost of the lowest specificity (15.87 ± 5.71%) of all the techniques analyzed. This is confirmed by receiver–operator curve analysis, as area under curve (AUC) was largest in SS (AUC for SS, Act80, Act40, Act20, MTN-B, and MTN-W were: 0.64, 0.15, 0.26, 0.38, 0.44, and 0.46, respectively).

**Table 2:** **Cross-modal comparison of sensitivities and specificities**.

	SS	Act80	Act40	Act20	MTN-B	MTN-W
Sensitivity	92.43 ± 1.83	93.00 ± 0.81	87.27 ± 1.30	80.00 ± 1.95	77.71 ± 3.27	59.50 ± 3.71
Specificity	69.69 ± 4.95	15.87 ± 3.04	30.23 ± 4.00	47.34 ± 5.52	56.65 ± 6.29	76.83 ± 5.71

*The sensitivities and specificities of sleep epoch detection are compared among the methods. SS performance was superior among the methods analyzed*.

*SS, SleepScope*.

**Figure 5:**
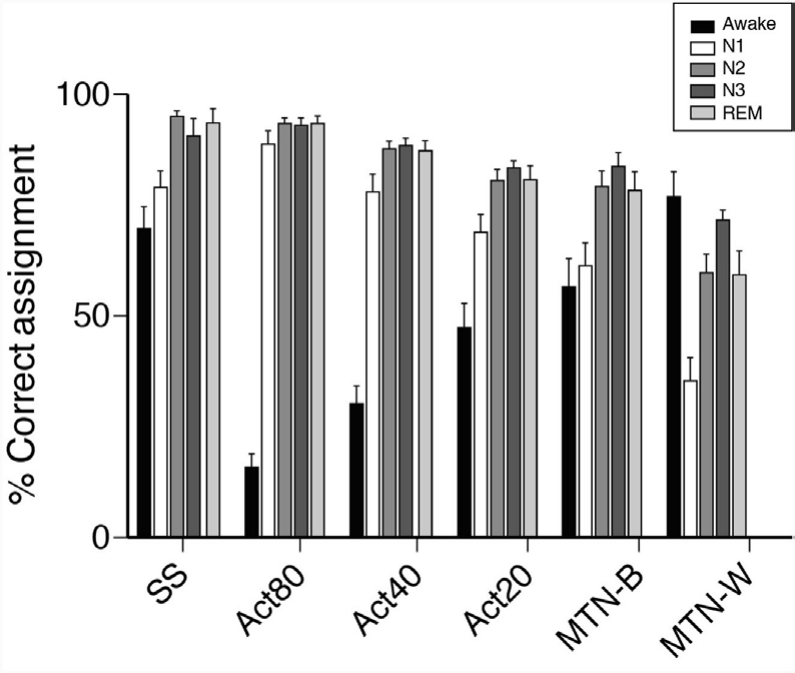
**Comparisons of epochs ratio, correctly assigned to sleep or awake**. Bar plots represent percentage of epochs correctly assigned to either sleep or awake are shown. Sleep/wake detection made by portable devices are examined in each PSG-defined sleep stages. In awake, % of epochs correctly detected by portable devices as awake are shown. In other sleep stages, % of epochs correctly detected by portable devices as sleep are shown.

Since activity-based methods demonstrated inferior sensitivity and specificity, the sleep stage-dependent accuracy of each technique was compared to explore the characteristics that compromised their estimations. With regard to sleep stage-based analysis, we found that estimations were poor at the awake and N1 stage compared to other sleep stages using the same method (Figure 5). These data demonstrated relatively poor performance of activity-based sleep monitors largely due to the misjudgment of N1 and wake stage epochs.

### Sleep Architecture Analysis by Single Channel EEG

Since the analysis indicated that SS results were most closely linked to PSG, the results were examined using additional data (30-s epoch data). These data demonstrated that the total sleep time estimated using SS did not significantly differ from PSG analysis (408.56 ± 10.22 vs. 403.88 ± 10.54 min for PSG and SS, respectively, *P* = 0.244 two-tailed paired Student’s *t*-test). Moreover, the correlation coefficient was strong (correlation coefficient: 0.93, *P* < 0.01), and Bland–Altman analysis confirmed the comparable assessment between PSG and SS, with negligible bias (bias ± SD was −4.68 ± 18.35, where 95% limits of agreement were −40.66 to 31.29). Upon combining these data, SS appeared to represent a good substitute for PSG.

Next, the ability of SS to estimate sleep stage duration was examined. The comparison of non-REM sleep Stage 1 (NS-1) lengths indicated that this estimation was comparable to that of PSG results (PSG: 44.60 ± 6.17 min vs. SS: 43.52 ± 3.90 min, *P* = 0.834 two-tailed paired Student’s *t*-test). However, the comparison of additional stages found differential results, since longer Stage 2 (PSG: 217.91 ± 11.40 min vs. SS: 238.03 ± 9.94 min, *P* < 0.01 two-tailed paired Student’s *t*-test), shorter Stage 3 (PSG: 69.96 ± 4.63 min vs. SS: 32.59 ± 6.62 min, *P* < 0.001 two-tailed paired Student’s *t*-test), and longer REM durations (PSG: 75.26 ± 6.18 min vs. SS: 89.74 ± 5.70 min, *P* < 0.01 two-tailed paired Student’s *t*-test) were observed using SS analysis. Sleep stage-wise agreement between PSG and SS were assessed by calculating percentage agreement of detected 30-s epochs (Table 3). This analysis showed good agreement (the Kappa statistics; *k* = 0.64 ± 0.03) between PSG and SS, with poorest performance for Stage 1 (30.56 ± 2.64% agreement) followed by awake detection (56.04 ± 4.49% agreement), whereas comparable performance for Stage 3, Stage 2, and REM. Despite a significant difference in the estimation of stage duration, the correlation between estimations and their corresponding PSG data indicated a moderate to strong correlation (correlation coefficient and *P* value for N1 were 0.57 and 0.0028, N2: 0.851 and <0.0001, N3: 0.56 and 0.0036, and REM: 0.75 and <0.0001, respectively). Bland–Altman analysis also indicated negligible bias for N1 assessment (−1.08 ± 23.86, −47.84 to 45.68) but systematic bias toward a decrease in N3 (−37.37 ± 26.21, −88.74 to 13.99) and an increase in SS for N2 (20.13 ± 28.09, −34.93 to 75.18) and REM (14.48 ± 19.89, −24.51 to 53.47). On combining these data, it was suggested that the systematic bias contributed to the differences in group-wise comparison, although the correlations were consistently strong, regardless of sleep stage.

**Table 3:** **Sleep stage-wise agreement between SS and PSG**.

		PSG				
		Awake	N1	N2	N3	REM
SS	Awake	56.05 ± 4.49	15.59 ± 2.26	3.23 ± 0.95	5.55 ± 2.74	10.4 ± 3.66
	N1	14.97 + 2.39	30.56 ± 2.64	6.1 ± 1.22	7.82 ± 3.6	7.59 ± 1.85
	N2	17.17 ± 3.15	43.55 ± 3.85	69.22 ± 2.64	15.85 ± 4.48	17.61 ± 5.17
	N3	6.98 ± 2.03	3.31 ± 1.93	17.32 ± 1.9	69.97 ± 8.58	2.57 ± 1.57
	REM	4.83 ± 1.29	6.98 ± 1.76	4.13 ± 1.29	0.81 ± 0.58	61.82 ± 7.32

*Sleep stage detection agreements between SS and PSG were compared. Within each sleep stage defined by PSG, % of epochs detected by SS as corresponding sleep stages were shown. Data are shown as mean ± SE in percentage. In this analysis, 30-s epochs were used to ensure higher resolution comparison*.

## Discussion

In this cross-modal comparison study, the EEG-based sleep monitor “SleepScope” was found to be superior to activity-based sleep monitors in accurately detecting sleep status.

As the representative activity-based sleep monitor, the widely used Actiwatch was compared against the newly developed MTN-210 device. Recently, multiple comparisons between wearable activity monitors drew attention, particularly in two categories; those of updated version of research level device or those of affordable consumer level devices (16, 27–29).

In terms of sleep detection, Ferguson and colleagues tested 7 consumer level activity recorders over 48 h of activity recordings in 21 healthy participants (15). They reported strong validity of these devices for sleep duration estimation (bias: 15.9–44.2 min, Pearson’s *r*: 0.82–0.92). Similarly, Rosenberger and colleagues conducted an examination using nine wearable activity monitors and concluded that they could be used as sleep length monitors at certain level of accuracy (mean error from 8.1 to 16.9%) (16).

However, these studies left several points to be addressed. First, these studies did not examine the specification of activity sensors that is necessary to isolate the mechanical potential of these devices. Second, these studies assessed gross sleep duration rather than sleep/wake detection accuracy and were therefore inadequate for the assessment of epoch-by-epoch accuracy of these devices. The other factor is that these studies employed “research level” activity or portable reduced-channel EEG monitors as reference, and therefore sleep might not be as accurately assessed as by PSG.

With respect to the mechanical aspect of activity sensors, both MTN-210 and Actiwatch featured a sensitivity preference range for activity above 75 rpm, a range that does not encompass the majority of sleep-related activities. This finding raised the possibility that future devices with a suitable detection range for sleep-related activity might be capable of more accurate sleep/wake delineation.

Direct comparison of activity counts between MTN-210 and Actiwatch demonstrated unpredicted results. Even with regard to differences in sensitivity and resolution, non-parametric testing confirmed that the strength of activity recorded by the MTN-210 and Actiwatch were closely related. Taken together, these findings suggest that MTN-210 could be used as an alternative life activity recorder in place of the Actiwatch.

In studies using “research level” activity monitors, it is indicated that activity-based methods feature limited performance, wherein good sleep detection sensitivity can only be achieved if specificity is compromised. For example, one of the largest study conducted by Kushida, in which 100 sleep disorder patients had participated, it was reported that sleep time, sleep efficiency, and even number of awakenings were successfully estimated by activity monitors (25). Similar findings were replicated in recent study in different populations. One study targeting 12 preschool children reported good performance of activity monitors in estimation of sleep latency, total sleep time, and sleep efficiency (30). However, even correlation analysis showed good performances in these studies; it has been noted that sleep detecting specificity is poor (31). Low sleep detecting specificity is more problematic when sleep is more fragmented (32). Therefore, the present study assessed both sensitivity and specificity to compare performance in addition to correlation analysis.

In this study, it was shown that SS and Act80 demonstrated good performance with regard to correlation analysis, although only SS featured superior performance in sensitivity and specificity analysis. The contrast of poor sleep estimation by Act40 and Act20 analysis and good sleep estimation by Act80 suggested a significant effect for the algorithm threshold. However, as a result of the high sensitivity to sleep detection, Act80 possesses a limited ability to detect wakefulness, especially when transitions with sleep epochs occur. Although this tendency is prominent in Act80, poorer performance rate in wake and N1 stage irrespective to methods which is consistent with previous reports (33, 34). Therefore, better algorithm that can detect the transition between wake and N1 stages should be the primary focus for the future development of ambulatory sleep recorders. Also, either analysis or data download was failed in one of the original 22 participant recordings in both MTN and Actiwatch. Although different cause was found for each case, mechanical reliability of the device system should be improved in the future.

In the current study, SleepScope estimation was the most accurate, among all the devices tested. This is evident from the high correlation of sleep length estimation and high AUC for sleep epoch detection, which was not observed when testing activity-based sleep monitors. In addition, unlike activity-based sleep monitors, EEG-based sleep monitors were able to assess sleep architecture, if not fully compatible with PSG. In particular, SleepScope uses the channel between forehead and mastoid, which is more susceptible to eye movements. This choice of electrodes could be advantageous for SleepScope to detect REM, which is not readily distinguishable from quiet wake by EEG alone. Even with regard to systematic bias (i.e., shorter estimation of N3 as well as longer estimation of N2 and REM), the advantage of EEG-based sleep monitors was that the levels of sleep/wake delineation were very similar to that obtained using PSG. Since systemic bias was the major issue with regard to SleepScope estimation, it is conceivable that a slight tuning of the EEG determination algorithm might reduce this bias. Since automatic sleep analysis algorithms are still under intensive development (17, 19–22), and the detailed information of SleepScope algorithm is not available, it is expected to develop open-source algorithm with multicenter validation.

In brief, EEG-based sleep monitoring should be the preferred method for future studies, especially in experiments that require measurement of naturalistic sleep quality. While alternatives that are more economical are available, activity-based sleep monitors produce less reliable data than single-channel EEG-based devices.

To the best of our knowledge, the current study is the first report that systemically compares portable devices with different modalities, EEG and activity counts, using an EEG oriented device, and activity-based sleep monitor, respectively. The effects of placement site were also investigated in the present experiment.

The current study features several limitations. Since participants mostly included university students, these results cannot be generalized to a larger more heterogeneous population or patient with sleep disorders. Also, caution should be taken in interpreting current results, as the participant number of current study was relatively small to conclude realistic conclusions. In addition, 2-min epochs we used in this study are not conventional in sleep research. Thus, it is difficult to directly compare current results to previous results, which are based on most conventional 30-s epochs. Although it might not directly affect inter-method comparisons, the so-called “first-night effect” might interfere with the current results, since PSG recordings were conducted on the first night in the PSG unit for all subjects. Also, in this study, a single examiner scored the PSG recordings. Therefore, the differences detected might have arisen partly due to the examiner’s preference.

In summary, the current study found that the newly developed affordable activity-based sleep monitors could be used as a substitute for the current standard Actiwatch. Moreover, single-channel EEG-based devices demonstrate superior performance compared to activity-based sleep recorders. These findings indicate that single-channel EEG-based devices might pose a novel technique for the assessment of sleep-related activity in a home-based rather than a laboratory setting.

## Author Contributions

HK and MM conceived the study. FM, MT, and MO recruited participants and conducted the study, under management by NY. KF conducted the mathematical analysis, and YS conducted the statistical analysis. TK scored PSG data.

## Conflict of Interest Statement

The authors declare that the research was conducted in the absence of any commercial or financial relationships that could be construed as a potential conflict of interest.
