# Supplementary material for: Comparisons of Portable Sleep Monitors of Different Modalities: Potential as Naturalistic Sleep Recorders
Source: Front Neurol. 2016 Jul 15;7:110. doi: 10.3389/fneur.2016.00110 (PMC4946159; doi:10.3389/fneur.2016.00110)
Supplement: Supplementary file 1 [file Table_1.PDF]

Supplementary Table 1: Stage-wise comparison of device ability

Numerical presentation of data shown in Figure 5. The percentage of correctly detected epochs are displayed for each method and sleep stage. Note that sleep epoch detecting performance of N1 stage or wake epoch detection in wake were lower than other stages, irrespective of method.

	SS	Act80	Act40	Act20	MTN-B	MTN-W
Wake	69.69±4.95	15.87±3.04	30.23±4.00	47.34±5.52	56.65±6.29	76.83±5.71
N1	78.98±3.75	88.8±2.96	77.92±4.01	68.87±3.99	61.33±5.20	35.36±5.23
N2	95.08±1.21	93.45±1.30	87.7±1.75	80.55±2.46	79.19±3.48	59.75±4.22
REM	93.57±3.20	93.45±1.72	87.33±2.22	80.74±3.15	78.31±4.2	59.26±5.41
SWS	90.66±3.91	93.04±1.71	88.48±1.64	83.36±1.63	83.73±3.17	71.63±2.22
